# Circ_0002715 promotes the development of osteoarthritis through regulating LXN by sponging miR-127-5p

**DOI:** 10.1186/s13018-023-03638-3

**Published:** 2023-03-22

**Authors:** Hongbo Liu, Hongxia Zhao, Yin Huang, Ming Lei

**Affiliations:** 1 Department of Rehabilitation, Chongqing Traditional Chinese Medicine Hospital, No. 6, Panxi Qizhi Road, Jiangbei District, Chongqing, 400021 China; 2 Department of Acupuncture, Chongqing Traditional Chinese Medicine Hospital, Chongqing, 400021 China

**Keywords:** Osteoarthritis, circ_0002715, miR-127-5p, LXN

## Abstract

**Background:**

Our study aims to investigate the role and mechanism of circular RNA_0002715 (circ_0002715) in osteoarthritis (OA) progression.

**Methods:**

IL-1β-induced CHON-001 cells were used to mimic OA cell model. Circ_0002715, microRNA (miR)-127-5p and Latexin (LXN) expression was detected by quantitative real-time PCR. Cell functions were determined by MTT assay, flow cytometry and ELISA assay. Protein expression was examined by western blot.

**Results:**

Circ_0002715 was highly expressed in OA cartilage tissues. Circ_0002715 silencing inhibited inflammation, apoptosis, and ECM degradation in IL-1β-interfered CHON-001 cells. Circ_0002715 could sponge miR-127-5p, and miR-127-5p could target LXN. The effect of circ_0002715 down-regulation on chondrocyte injury was partially restored by miR-127-5p inhibitor. MiR-127-5p could suppress chondrocyte injury by inhibiting LXN expression.

**Conclusion:**

Circ_0002715 might be a new therapeutic target for OA, which regulated miR-127-5p/LXN axis to promote IL-1β-induced chondrocyte injury.

**Supplementary Information:**

The online version contains supplementary material available at 10.1186/s13018-023-03638-3.

## Background

Osteoarthritis (OA) is mainly caused by degeneration of articular cartilage and reduction of cartilage matrix [1, 2]. The pain and disability caused by OA pose a serious threat to the health of older people [3, 4]. More and more studies have confirmed that chondrocyte apoptosis, inflammation and ECM degradation are important causes of articular cartilage lesions [5, 6]. Therefore, finding the molecular mechanism that can effectively regulate chondrocyte apoptosis, inflammation and ECM degradation is helpful to provide a potential molecular target for OA treatment. IL-1β is the main pro-inflammatory cytokine present in the cartilage of arthritis, and its induced chondrocytes have been widely used to construct OA models in vitro at present [7, 8].

Circular RNAs (circRNAs) are closed non-coding RNA molecules without 3′ and 5′ ends [9]. Many circRNAs have been discovered to play a role in regulating chondrocyte functions in OA, such as circ-IQGAP1 and circ_0002715 [10, 11]. Luo et al*.* identified a significant increase in circ_0002715 in the peripheral blood of rheumatoid arthritis patients [12]. However, the function of circ_0002715 in OA development is not clear. This study selected circ_0002715 to explore its role in OA progression.

MicroRNAs (miRNAs) are short (22 nucleotide length) non-coding RNA molecules that can be adsorbed by circRNAs to regulate the expression of targeted mRNAs [13, 14]. Many studies have shown that miRNA and gene is involved in the regulation of tendon homeostasis, and their expression is related to the pathogenesis of tendon injury [15, 16]. Many miRNAs play important roles in OA progression [17–19]. MiR-127-5p is low expressed in OA cartilage, which can regulate MMP13 or osteopontin (OPN) to mediate OA development [20, 21].

Latexin (LXN), the first to be found in the rat neocortex, is the only known endogenous inhibitor of carboxypeptidase [22, 23]. Recently, numerous studies have found that abnormal expression of LXN can cause inflammatory diseases in vivo, such as colitis and acute pancreatitis [24, 25]. In OA-related studies, LXN had been shown to be highly expressed at the early stage of OA and was associated with articular cartilage mineralization [26, 27]. Importantly, LXN a downstream regulator of the circ_0094742/miR-127-5p axis, has been reported to mediate OA development by regulating chondrocyte viability [28]. Therefore, LXN may be an important regulator of OA progression.

This study aims to explore the role and mechanism of circ_0002715 in OA progression. Through bioinformatics analysis, we found that circ_0002715 sponged miR-127-5p, and miR-127-5p could bind with LXN 3′UTR. Therefore, our study proposed the hypothesis that circ_0002715 regulates OA progression through miR-127-5p/LXN.

## Methods

### Patients and cell lines

Cartilage tissues from 30 patients with knee OA at Chongqing Traditional Chinese Medicine Hospital were collected. Normal cartilage tissue (n = 30) was obtained during surgery in age-matched fracture patients with no symptoms of OA. This study was approved by the Ethics Committee of Chongqing Traditional Chinese Medicine Hospital.

CHON-001 cells (ATCC, Manassas, VA, USA) were stimulated with 10 ng/mL IL-1β (Amyjet, Wuhan, China) for 24 h to mimic OA cell model.

### Cell transfection

Short interfering RNA (siRNA) targeting circ_0002715 (si-circ_0002715^#1/2/3^), circ_0002715 overexpression vector, miR-127-5p mimic, inhibitor (in-miR-127-5p), siRNA targeting LXN (si-LXN), LXN overexpression vector, and their matched controls (Ribobio, Guangzhou, China) were transfected into cells using Lipofectamine 3000.

### Quantitative real-time PCR (qRT-PCR)

Total RNAs were isolated by Trizol Reagent and then reverse transcribed into cDNA with Reverse Transcription Kit. QRT-PCR was performed using SYBR Green (Takara, Dalian, China) and specific primers (Table 1). Relative expression was calculated by 2^−ΔΔCT^. RNase R digestion, RNA was incubated with RNase R solution and then used for qRT-PCR.

**Table 1:** The primer sequences for qRT-PCR

Name		Primers (5′-3′)
circ_0002715	Forward	CAAACCTCCTCTCCATGCTC
	Reverse	GCACTCTGAAGCCGAAGTGT
miR-127-5p	Forward	GCCGAGCTGAAGCTCAGAGG
	Reverse	CAGTGCGTGTCGTGGAGT
LXN	Forward	ACAGAACTACATCAACTACCAGC
	Reverse	GTGATACTTATGTCCTCTTCCTGG
β-actin	Forward	CTCGCCTTTGCCGATCC
	Reverse	TCTCCATGTCGTCCCAGTTG
U6	Forward	CTCGCTTCGGCAGCACA
	Reverse	AACGCTTCACGAATTTGCGT

### Cellular distribution analysis

RNAs were extracted from the cytoplasmic and nuclear sections of CHON-001 cells using Paris kits (Thermo Fisher, Waltham, MA, USA). QRT-PCR was performed to detect circ_0002715 level in cytoplasm RNA or nuclear RNA with β-actin U6 or as cytoplasm reference or nuclear reference, respectively.

### MTT assay

CHON-001 cells seeded in 96-well plates were cultured for indicated time points. Cell was incubated with MTT reagent (BeyoTime, Shanghai, China) and hatched with DMSO. The absorbance at 490 nm was recorded by microplate reader (Multiskan SkyHigh, Thermo Fisher) to assess cell viability.

### Flow cytometry assay

The transfected cells were suspended with binding buffer and incubated with Annexin-FITC and PI (BeyoTime). Apoptotic cells were detected by flow cytometry (FACSCalibu).

### ELISA assay

The medium of CHON-001 cells was collected and applied to detect the contents of IL-6, IL-8, and TNF-α using corresponding ELISA kit.

### Western blot analysis

Total proteins were separated using RIPA buffer (RIPA; Thermo Fisher). Protein samples were loaded into SDS-PAGE gel and transferred to PVDF membranes. Membranes were incubated with anti-β-actin, anti-Bcl2, anti-cleaved-caspase-3, anti-p21, anti-CyclinD1, anti-MMP-13, and anti-MMP-3. Then, membrane was treated with secondary antibody, and protein signals were visualized by BeyoECL Plus kit (BeyoTime).

### Dual-luciferase reporter assay

The sequences of circ_0002715 and LXN 3′UTR were inserted into pmirGLO reporter vectors to generate the circ_0002715-WT/MUT vectors and LXN 3′UTR-WT/MUT vectors, respectively. HEK293T cells were co-transfected with the above vectors and miR-127-3p mimic/miR-NC. 48 h later, luciferase activity was analyzed by corresponding Kit (BeyoTime) with microplate reader (Multiskan SkyHigh, Thermo Fisher).

### RIP assay

CHON-001 cell lysate was incubated with magnetic beads-conjugated with human anti-Ago2 or anti-IgG. Relative enrichments were determined by qRT-PCR analysis.

### Statistical analysis

Data were shown as the mean ± SD. Comparison was determined using Student’s *t*-test or ANOVA. GraphPad Prism 7 was utilized for data analysis. *P* < 0.05 meant significant difference.

## Results

### Circ_0002715 expression in cartilage tissues and IL-1β-induced chondrocytes

**Fig. 1:**
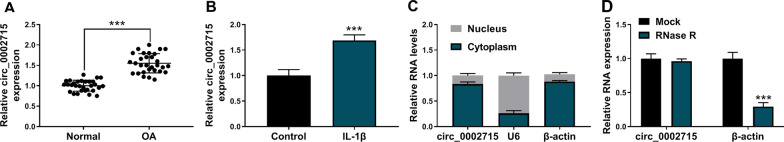
Circ_0002715 was up-regulated in cartilage tissues and IL-1β-induced chondrocytes. **A**, **B** Circ_0002715 expression was tested by qRT-PCR. **C** The distribution of circ_0002715 in nucleus or cytoplasm was determined by qRT-PCR. **D** RNase R was used to assess the resistance of circ_0002715 on RNase R digestion. ***P < 0.001

Circ_0002715 was significantly enhanced in cartilage from OA patients and IL-1β-induced CHON-001 cells (Fig. 1A, B). Circ_0002715 was mainly distributed in the cytoplasm by cell localization (Fig. 1C). After RNase R treatment, we confirmed that circ_0002715 could resist the digestion of RNase R (Fig. 1D).

### Circ_0002715 silencing inhibited IL-1β-induced chondrocyte injury

**Fig. 2:**
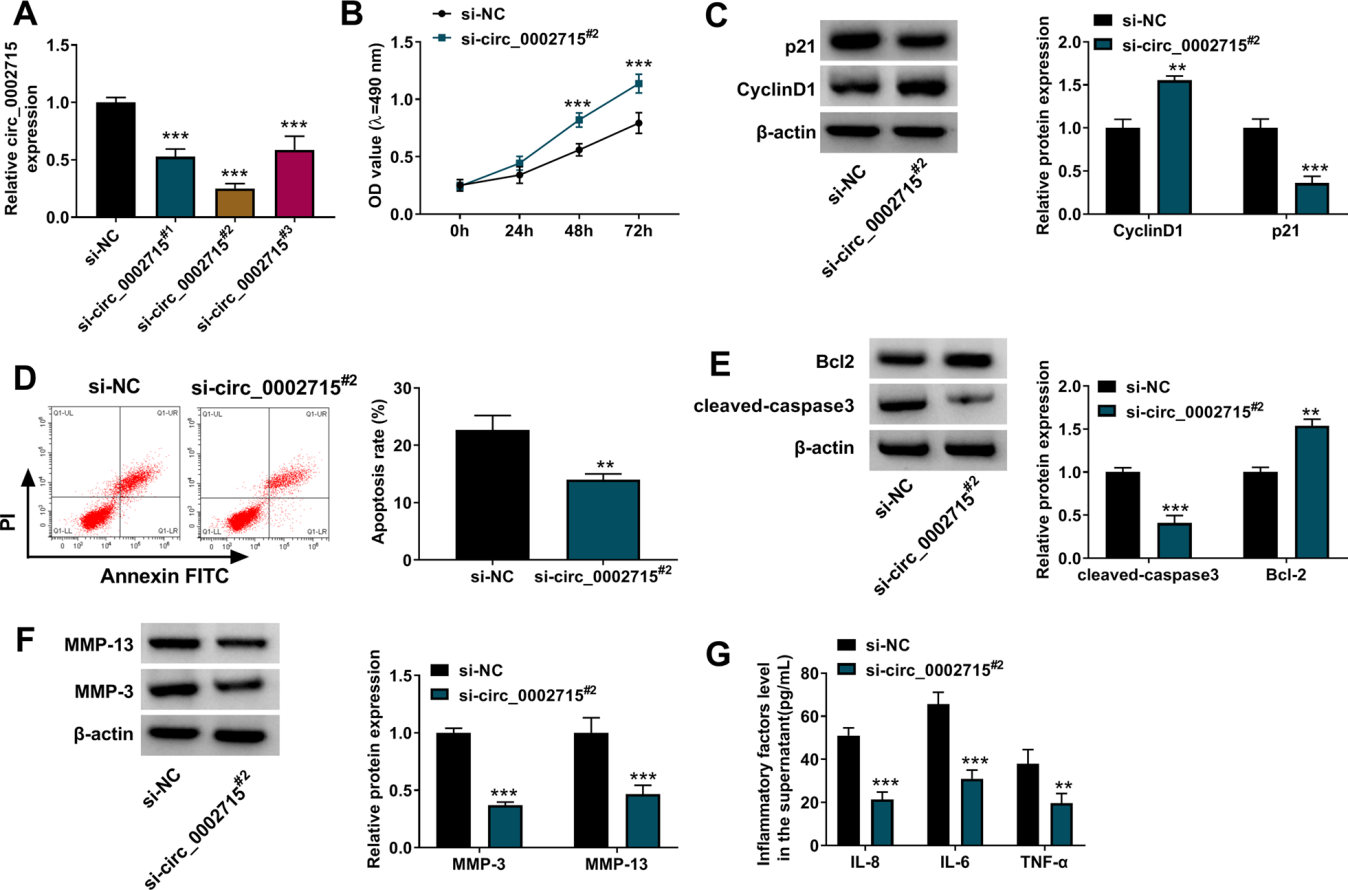
The functional roles of circ_0002715 in IL-1β-induced CHON-001 cells. **A** The expression level of circ_0002715 was determined by qRT-PCR. **B** MTT assay. **C** Western blot. **D** Flow cytometry. **E**, **F** Western blot. **G** ELISA. **P < 0.01, ***P < 0.001

To explore the role of circ_0002715 in OA, chondrocytes were transfected with the siRNA for circ_0002715 followed by treated with IL-1β. As shown in Fig. 2A, si-circ_0002715^#2^ had the best knockdown effect on circ_0002715 expression, so subsequent experiments all adopted si-circ_0002715^#2^. Silenced circ_0002715 significantly promoted cell viability (Fig. 2B). Besides, cell cyclin marker CyclinD1 protein expression was enhanced, while cell cycle inhibitor p21 protein expression was reduced after circ_0002716 knockdown (Fig. 2C). Circ_0002715 silencing significantly inhibited the apoptotic rate of IL-1β-induced chondrocytes (Fig. 2D). Also, si-circ_0002715^#2^ significantly increased Bcl-2 protein level, while decreased the protein levels of cleaved-caspase 3, MMP-13, and MMP-3 (Fig. 2E, F). The levels of inflammatory factors were down-regulated by si-circ_0002715^#2^ in IL-1β-induced chondrocytes (Fig. 2G).

### Circ_0002715 acted as a sponge for miR-127-5p

**Fig. 3:**
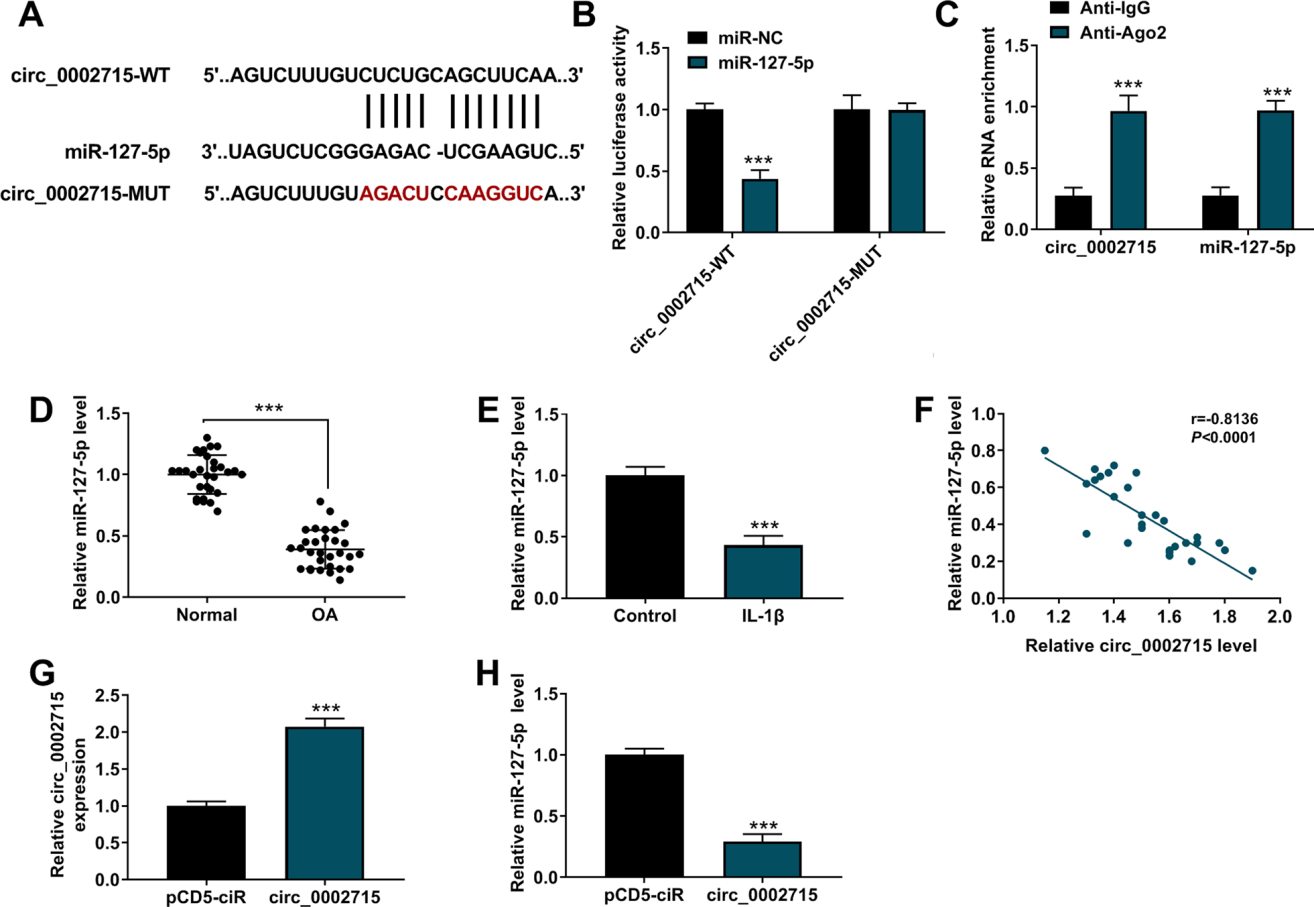
Circ_0002715 functioned as a sponge of miR-127-5p. **A** The complementary sequences between miR-127-5p and circ_0002715 were shown. **B** Dual-luciferase reporter assay. **C** RIP assay. **D**, **E** The expression of miR-127-5p in cartilage tissues and IL-1β-induced chondrocytes was tested by qRT-PCR. **F** Pearson’s correlation analysis. **G**, **H** QRT-PCR was employed to detect the expression of circ_0002715 and miR-127-5p. ***P < 0.001

MiR-127-5p had complementary binding sites with circ_0002715 predicted by circinteractome software (Fig. 3A). MiR-127-5p overexpression could only inhibit the luciferase activity of circ_0002715-WT reporter vector (Fig. 3B). RIP test further confirmed the direct interaction between miR-127-5p and circ_0002715 (Fig. 3C). MiR-127-5p expression was significantly reduced in both OA cartilage tissues and IL-1β-induced CHON-001 cells (Fig. 3D, E). Circ_0002715 expression was negatively correlated with miR-127-5p expression in OA cartilage tissues (Fig. 3F). After confirmed the over-expression efficiency of circ_0002715 overexpression vector (Fig. 3G), we detected miR-127-5p expression and confirmed that circ_0002715 overexpression could reduce miR-127-5p expression (Fig. 3H).

### Circ_0002715 regulated IL-1β-induced chondrocyte injury through miR-127-5p

**Fig. 4:**
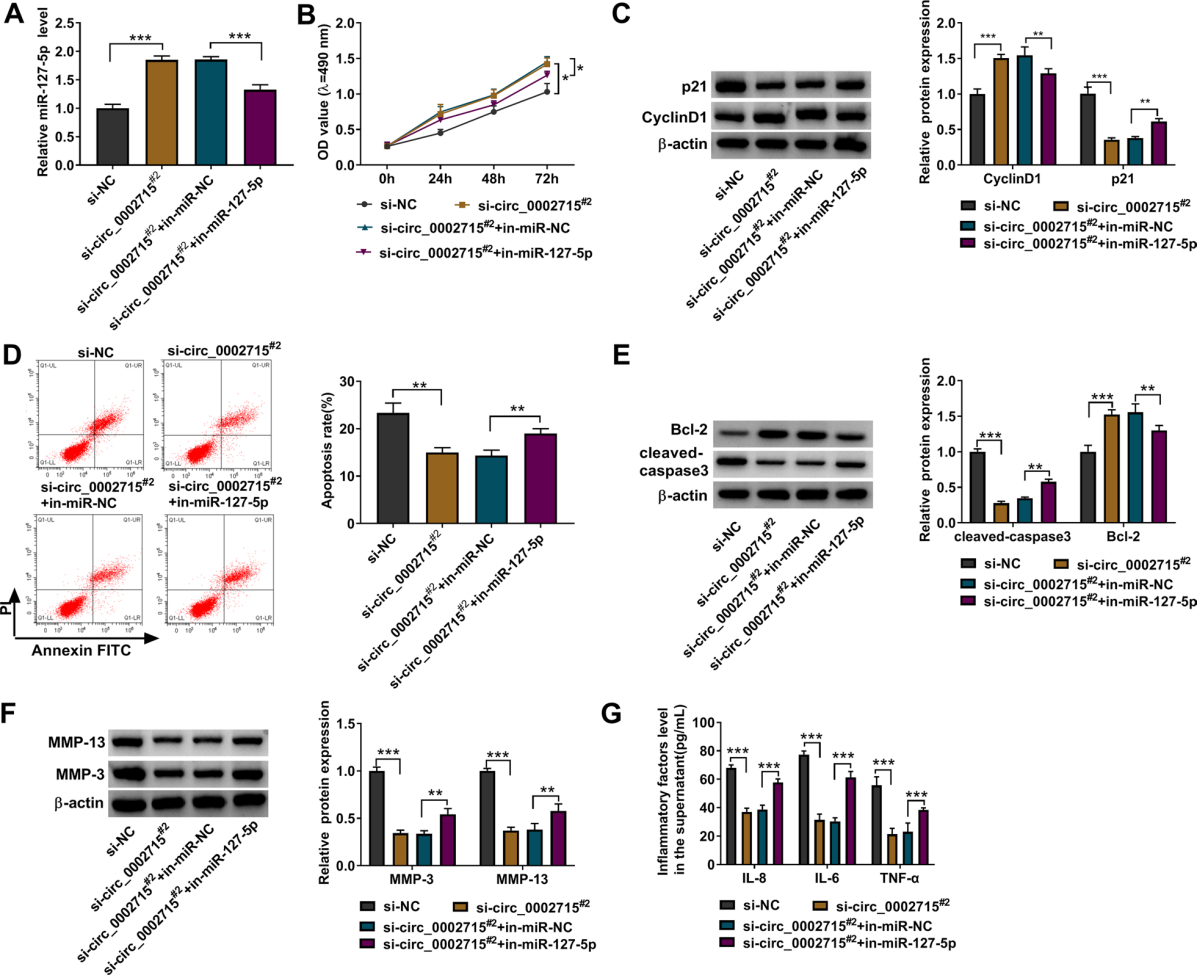
Circ_0002715 regulated the behavior of IL-1β-induced chondrocytes through miR-127-5p. **A** QRT-PCR detected the expression of miR-127-5p. **B** MTT assay. **C** Western blot analysis. **D** Flow cytometry. **E**, **F** Western blot. **G** ELISA assay. *P < 0.05, **P < 0.01, ***P < 0.001

Subsequently, chondrocytes were co-transfected with si-circ_0002715^#2^ and in-miR-127-5p followed by treated with IL-1β. As presented in Fig. 4A, in-miR-127-5p eliminated the promotion effect of si-circ_0002715^#2^ on miR-127-5p expression. MiR-127-5p inhibitor partially down-regulated the enhancing effect of si-circ_0002715^#2^ on cell viability (Fig. 4B). Also, the regulation of si-circ_0002715^#2^ on p21 and CyclinD1 protein expression was reversed by in-miR-127-5p (Fig. 4C). The inhibitory of circ_0002715 knockdown on cell apoptosis could be eliminated by in-miR-127-5p (Fig. 4D). Circ_0002715 silencing increased Bcl2 protein level, decreased the cleaved-caspase3, MMP-3 and MMP-13 protein levels, and reduced the levels of inflammation factors. However, these effects could be reversed by in-miR-127-5p (Fig. 4E–G). To further confirm our results, chondrocytes were co-transfected with circ_0002715 overexpression vector and miR-127-5p mimic followed by treated with IL-1β. MiR-127-5p mimic promoted miR-127-5p expression suppressed by circ_0002715 (Additional file 1: Fig. S1A). Overexpression of miR-127-5p partially reversed circ_0002715-mediated viability inhibition of IL-1β-induced chondrocytes (Additional file 1: Fig. S1B). As presented in Additional file 1: Fig. S1C, the effect of circ_0002715 on CyclinD1 and p21 protein expression was overturned by miR-127-5p. MiR-127-5p could restore the promotion of apoptosis by circ_0002715 in IL-1β-induced chondrocytes (Additional file 1: Fig. S1D). MiR-127-5p overexpression reversed the regulation of circ_0002715 on Bcl-2, cleaved-caspase3, MMP-3, and MMP-13 protein levels, as well as IL-8, IL-6 and TNF-α levels (Additional file 1: Fig. S1E–G).

### LXN was a direct target of miR-127-5p

**Fig. 5:**
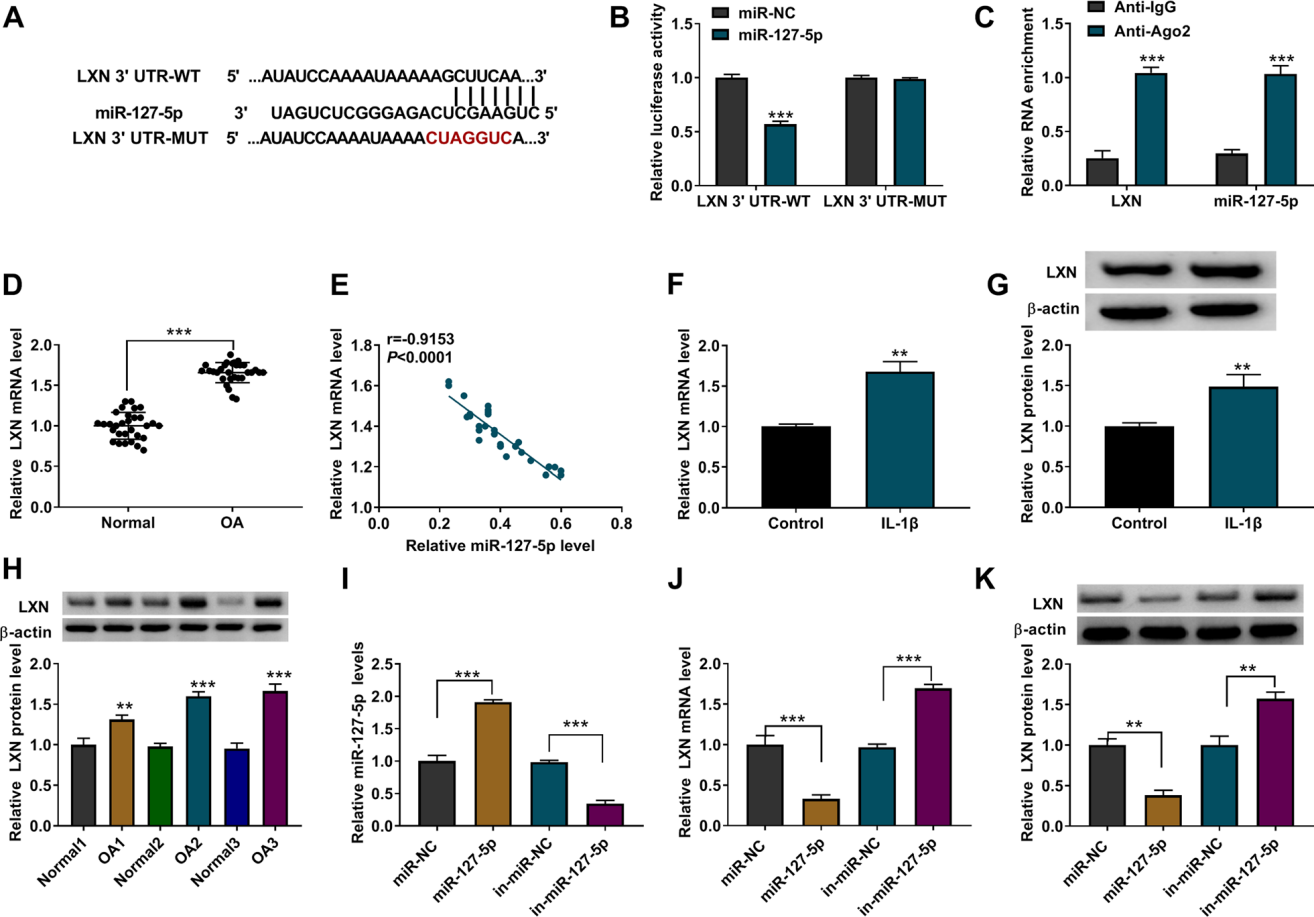
LXN was a target of miR-127-5p. **A** The complementary sequences between miR-127-5p and LXN 3′UTR were shown. **B** Dual-luciferase reporter assay. **C** RIP assay. **D**, **F** The expression of LXN in cartilage tissue and IL-1β-induced chondrocytes was tested by qRT-PCR. **E** Pearson’s correlation analysis. **G**, **H** Western blot was used to examine the expression of LXN in cartilage tissues and IL-1β-induced chondrocytes. **I** QRT-PCR was used to test assessed the transfection efficiency of miR-127-5p mimic or inhibitor. **J**, **K** QRT-PCR and western blot were performed to test LXN mRNA and protein levels. **P < 0.01, ***P < 0.001

Targetscan software predicted the binding sites between LXN 3′UTR and miR-127-5p (Fig. 5A). MiR-127-5p mimic inhibited the luciferase activity of LXN 3′UTR-WT vector (Fig. 5B). RIP test further confirmed the direct interaction between miR-127-5p and LXN (Fig. 5C). In OA cartilage tissues, LXN mRNA level was significantly increased and was negatively correlated with miR-127-5p level (Fig. 5D, E). Besides, LXN also had an elevated expression in IL-1β induced CHON-001 cells at the mRNA level and protein level (Fig. 5F, G). LXN protein expression was increased in OA cartilage tissues (Fig. 5H). After confirmed the efficiencies of miR-127-5p mimic and inhibitor (Fig. 5I), we discovered that LXN level was significantly decreased by miR-127-5p mimic, while increased by miR-127-5p inhibitor (Fig. 5J, K).

### MiR-127-5p regulated the behavior of IL-1β-induced chondrocytes through LXN

**Fig. 6:**
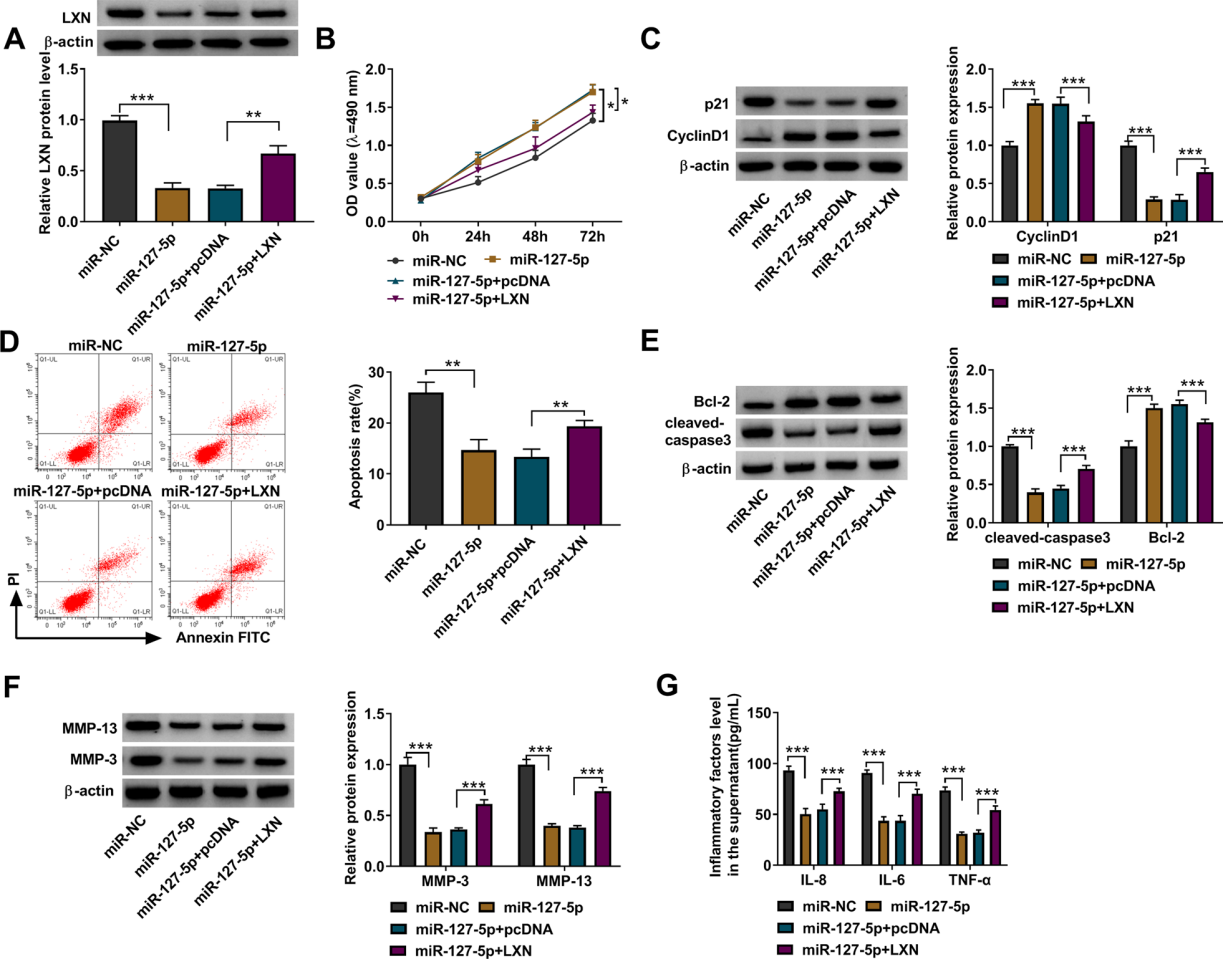
MiR-127-5p regulated the behavior of IL-1β-induced chondrocytes by LXN. **A** Western blot was performed to detect the expression of LXN. **B** MTT assay. **C** Western blot. **D** Flow cytometry. **E**, **F** Western blot. **G** ELISA assay. *P < 0.05, **P < 0.01, ***P < 0.001

The addition of pcDNA LXN overexpression vector significantly upregulated the protein level of LXN suppressed by miR-127-5p mimic (Fig. 6A). In functional experiments, LXN partially reduced the promotion effect of miR-127-5p on the viability and CyclinD1 protein level, while reversed the inhibition effect on p21 protein level (Fig. 6B, C). Similarly, LXN overexpression restored miR-127-5p-mediated apoptosis inhibition (Fig. 6D). MiR-127-5p enhanced Bcl-2 protein expression, while reduced cleaved-caspase3, MMP-3, and MMP-13 protein levels, and these effects were reversed by LXN overexpression (Fig. 6E, F). Also, overexpression of LXN could partially up-regulate inflammation factor levels, which were reduced by miR-127-5p (Fig. 6G). To further confirm our results, chondrocytes were co-transfected with in-miR-127-5p and si-LXN followed by treated with IL-1β. As shown in Additional file 2: Fig. S2A, si-LXN overturned the upregulation of in-miR-127-5p on LXN protein level. Silencing of LXN partially restored the regulation of in-miR-127-5p on cell viability, CyclinD1 and p21 protein expression (Additional file 2: Fig. S2B, C). Moreover, the promoting effect of miR-127-5p inhibitor on cell apoptosis could also be eliminated by LXN knockdown (Additional file 2: Fig. S2D). Furthermore, LXN silencing reversed the regulation of in-miR-127-5p on Bcl-2, cleaved-caspase3, MMP-3 and MMP-13 protein expression, as well as inflammation factor levels (Additional file 2: Fig. S2E–G).

### Circ_0002715 sponged miR-127-5p to target LXN

**Fig. 7:**
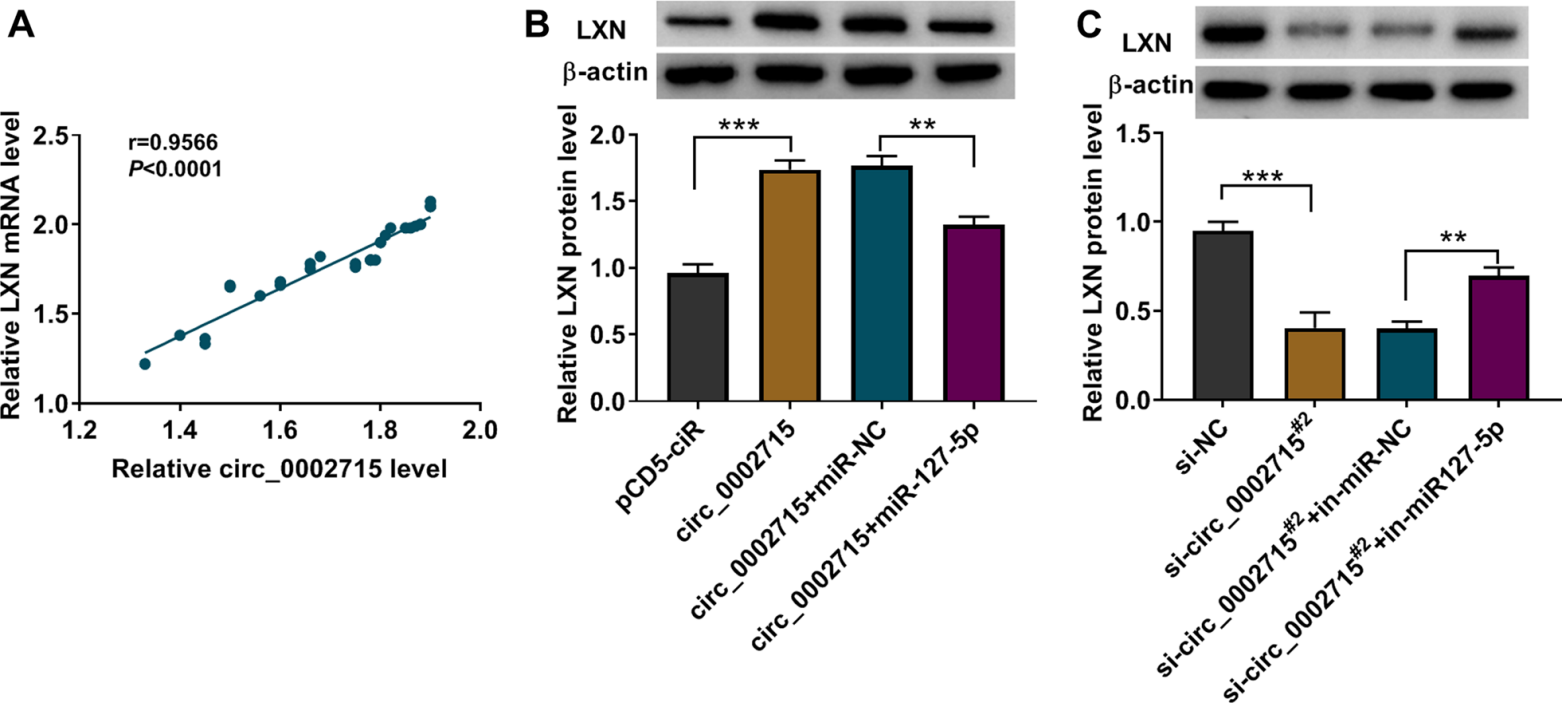
Circ_0002715 sponged miR-127-5p to target LXN. **A** Pearson’s correlation analysis. **B**, **C** Western blot tested the expression of LXN in transfected IL-1β-induced CHON-001 cells. **P < 0.01, ***P < 0.001

Circ_0002715 level was positively correlated with LXN level in OA cartilage tissues (Fig. 7A). MiR-127-5p mimic partially overturned the increasing effect of circ_0002715 overexpression on LXN protein level (Fig. 7B). Similarly, miR-127-5p inhibitor partially reversed the decreasing effect of circ_0002715 knockdown on LXN protein expression (Fig. 7C).

## Discussion

The etiology and pathogenesis of OA is very complicated, and its understanding is not very clear at present. Numerous studies have proved that OA may be caused by degenerative diseases of articular cartilage [29]. Here, we investigated the possible effect of circ_0002715 on OA by inducing inflammation, apoptosis and ECM degradation of chondrocytes with IL-1β. Consistent with previous studies [12], circ_0002715 was up-regulated in both OA cartilage tissues. Circ_0002715 aggravated IL-1β-induced CHON-001 cell injury. These data suggested that circ_0002715 might be an effective target for treating OA.

Numerous reports suggest that circRNA can act as miRNA sponge to regulate mRNA expression as competitive endogenous RNA (ceRNA) [30]. MiR-127-5p was found to be under-expressed in OA, and it could be targeted by circRNA.33186, circ_0136474 or circ_0094742 to regulate the function of OA chondrocytes [28, 31, 32]. Our results also proved that miR-127-5p expression was low in OA cartilage tissues, and circ_0002715 could serve as a ceRNA for miR-127-5p. MiR-127-5p contributed to IL-1β-infected CHON-001 cell injury. The rescue experiments suggested that the promotion effect of circ_0002715 on IL-1β-induced CHON-001 cell injury was abolished by miR-127-5p mimic. MiR-127-5p inhibitor also reversed the inhibition effect of circ_0002715 knockdown on IL-1β-induced CHON-001 cell injury. It was demonstrated that circ_0002715 promoted OA progression by targeting miR-127-5p.

LXN was elevated in OA cartilage tissues and it was predicted to be targeted by miR-127-5p. Functionally, LXN overexpression eliminated the inhibition effect of miR-127-5p overexpression on IL-1β-induced CHON-001 injury. And silencing of LXN partially restored the promotion effect of miR-127-5p inhibitor on CHON-001 cell injury. Circ_0002715 could positively regulate LXN expression by sponging miR-127-5p, further supporting the mechanism of circ_0002715/miR-127-3p/LXN.

## Conclusion

We found that circ_0002715 regulated miR-127-5p/LXN pathway, thus promoting chondrocyte inflammation, apoptosis and ECM degradation induced by IL-1β. These results provide evidence for the use of targeted circ_0002715/miR-127-5p/LXN axis in the treatment of OA, suggesting that targeted inhibition of circ_0002715 may be an effective strategy for OA treatment.

## Supplementary Information

**Additional file 1: Fig. S1**. Circ_0002715 and miR-127-5p regulated the behavior of IL-1β-induced chondrocytes. (A) The expression of miR-127-5p was detected by qRT-PCR. (B) MTT assay. (C) Western blot. (D) Flow cytometry. (E, F) Western blot. (G) ELISA assay. *P < 0.05, **P < 0.01, ***P < 0.001.

**Additional file 2: Fig. S2.** MiR-127-5p and LXN regulated the behavior of IL-1β-induced chondrocytes. (A) LXN protein expression was tested by western blot. (B) MTT assay. (C) Western blot. (D) Flow cytometry. (E, F) Western blot. (G) ELISA assay. *P < 0.05, **P < 0.01, ***P < 0.001.

## Data Availability

The datasets used and analyzed during the current study are available from the corresponding author on reasonable request.
