# Supplementary figures and images for: Circ_0002715 promotes the development of osteoarthritis through regulating LXN by sponging miR-127-5p
Source: J Orthop Surg Res. 2023 Mar 22;18:230. doi: 10.1186/s13018-023-03638-3 (PMC10031964; doi:10.1186/s13018-023-03638-3)

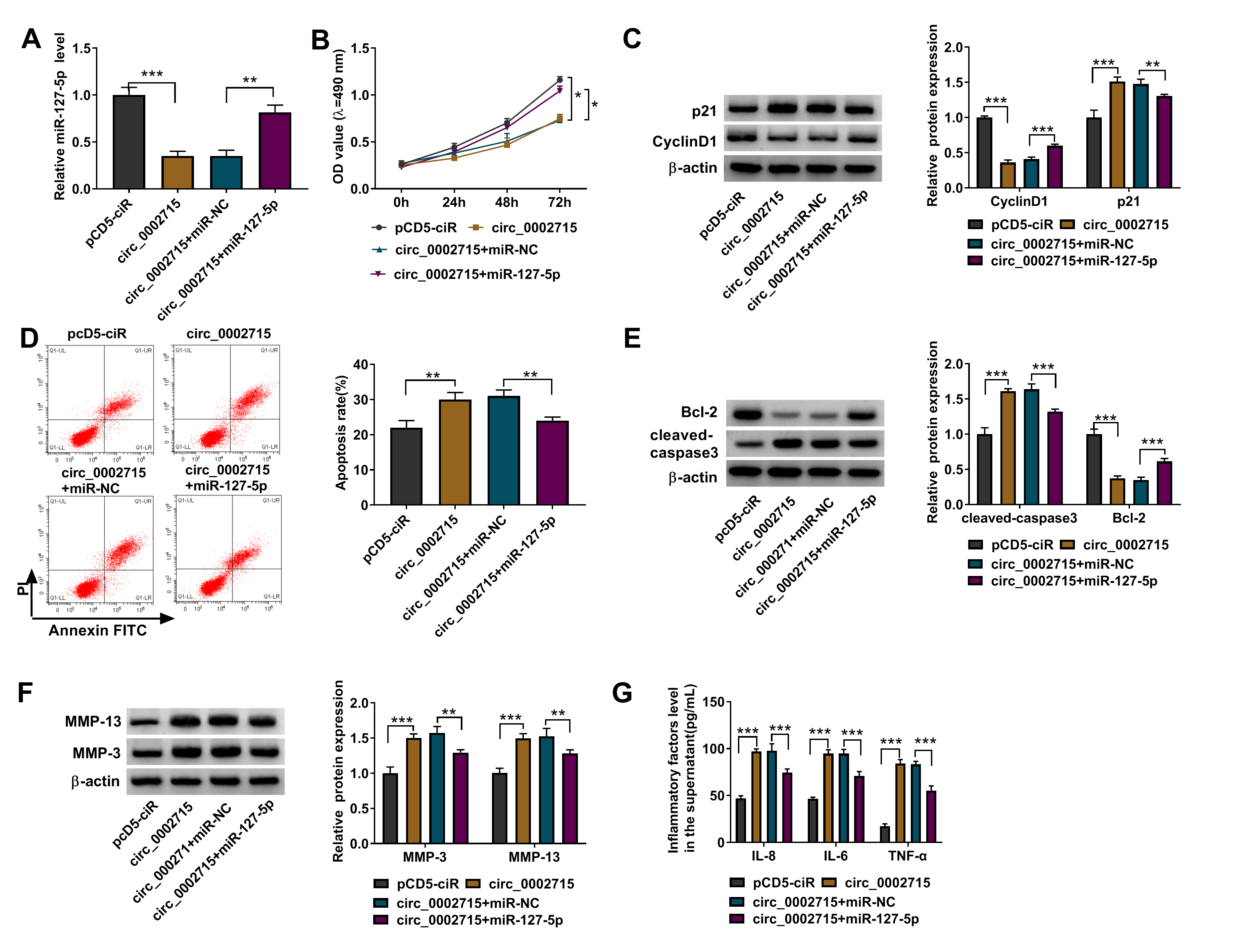

Supplement: Supplementary file 1 — Additional file 1: Fig. S1. Circ_0002715 and miR-127-5p regulated the behavior of IL-1β-induced chondrocytes. (A) The expression of miR-127-5p was detected by qRT-PCR. (B) MTT assay. (C) Western blot. (D) Flow cytometry. (E, F) Western blot. (G) ELISA assay. *P < 0.05, **P < 0.01, ***P < 0.001. [file 13018_2023_3638_MOESM1_ESM.tif]

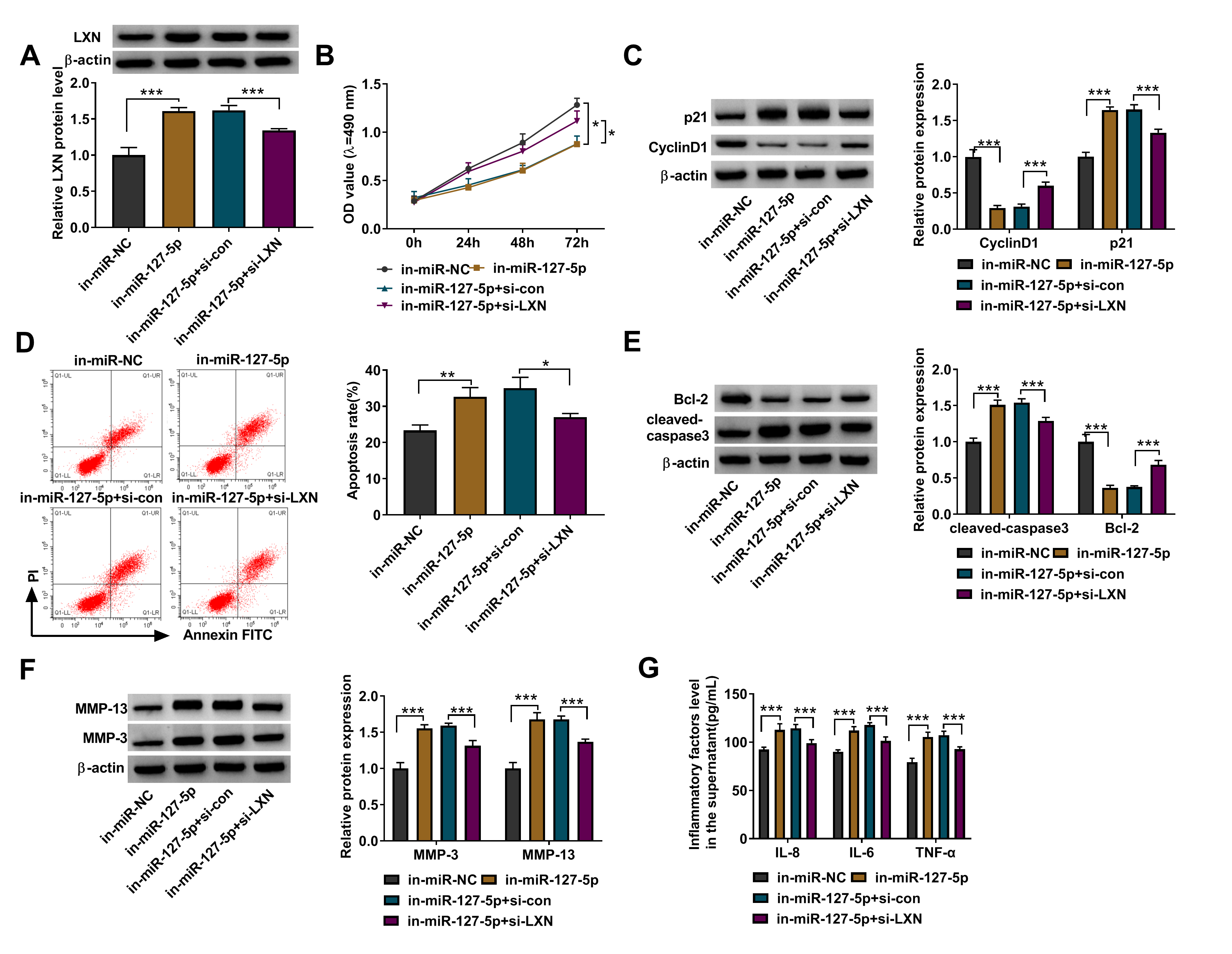

Supplement: Supplementary file 2 — Additional file 2: Fig. S2. MiR-127-5p and LXN regulated the behavior of IL-1β-induced chondrocytes. (A) LXN protein expression was tested by western blot. (B) MTT assay. (C) Western blot. (D) Flow cytometry. (E, F) Western blot. (G) ELISA assay. *P < 0.05, **P < 0.01, ***P < 0.001. [file 13018_2023_3638_MOESM2_ESM.tif]
